# Quantification system for the viral dynamics of a highly pathogenic simian/human immunodeficiency virus based on an *in vitro *experiment and a mathematical model

**DOI:** 10.1186/1742-4690-9-18

**Published:** 2012-02-25

**Authors:** Shingo Iwami, Benjamin P Holder, Catherine AA Beauchemin, Satoru Morita, Tetsuko Tada, Kei Sato, Tatsuhiko Igarashi, Tomoyuki Miura

**Affiliations:** 1 Precursory Research for Embryonic Science and Technology (PRESTO), Japan Science and Technology Agency (JST), Kawaguchi, Saitama 332-0012, Japan; 2 Graduate School of Mathematical Sciences, The University of Tokyo, Meguro-ku, Tokyo 153-8914, Japan; 3 Institute for Virus Research, Kyoto University, Kyoto, Kyoto 606-8507, Japan; 4 Department of Physics, Ryerson University, ON, Toronto M5B 2K3, Canada; 5 Department of Systems Engineering, Shizuoka University, Hamamatsu, Shizuoka 432-8561, Japan; 6 Department of Biology, Faculty of Sciences, Kyushu University, 6-10-1 Hakozaki, Higashi-ku, Fukuoka, Fukuoka 812-8581, Japan

**Keywords:** Viral infectiousness, Quantification of viral dynamics, *In vitro *experiment, Mathematical model, Simian/Human immunodeficiency virus

## Abstract

**Background:**

Developing a quantitative understanding of viral kinetics is useful for determining the pathogenesis and transmissibility of the virus, predicting the course of disease, and evaluating the effects of antiviral therapy. The availability of data in clinical, animal, and cell culture studies, however, has been quite limited. Many studies of virus infection kinetics have been based solely on measures of total or infectious virus count. Here, we introduce a new mathematical model which tracks both infectious and total viral load, as well as the fraction of infected and uninfected cells within a cell culture, and apply it to analyze time-course data of an SHIV infection *in vitro*.

**Results:**

We infected HSC-F cells with SHIV-KS661 and measured the concentration of Nef*-*negative (target) and Nef*-*positive (infected) HSC-F cells, the total viral load, and the infectious viral load daily for nine days. The experiments were repeated at four different MOIs, and the model was fitted to the full dataset simultaneously. Our analysis allowed us to extract an infected cell half-life of 14.1 h, a half-life of SHIV-KS661 infectiousness of 17.9 h, a virus burst size of 22.1 thousand RNA copies or 0.19 TCID_50_, and a basic reproductive number of 62.8. Furthermore, we calculated that SHIV-KS661 virus-infected cells produce at least 1 infectious virion for every 350 virions produced.

**Conclusions:**

Our method, combining *in vitro *experiments and a mathematical model, provides detailed quantitative insights into the kinetics of the SHIV infection which could be used to significantly improve the understanding of SHIV and HIV-1 pathogenesis. The method could also be applied to other viral infections and used to improve the *in vitro *determination of the effect and efficacy of antiviral compounds.

## Background

Historically, the study of the highly pathogenic simian/human immunodeficiency virus (SHIV) has provided important information for the understanding of human immunodeficiency virus type-1 (HIV-1) pathogenesis. For example, it was clarified in an SHIV animal study that co-receptor usage determined by the HIV-1 *env *gene affects the virus' cell tropism (preference for specific target cell populations), and thus its pathogenesis, *in vivo *[1-3]. Furthermore, infections with highly pathogenic SHIV strains in animal models have exhibited stable clinical manifestations in most infected animals, similar to an aspect of infection course in human HIV infections [4,5]. One of the highly pathogenic SHIV strains, SHIV-KS661, which has the *env *gene of HIV-1 89.6 and predominantly uses CXCR4 as the secondary receptor for its infection [2], causes an infection that systemically depletes the CD4^+ ^T cells of rhesus macaques within 4 weeks after infection [6,7]. In observations by our group in recent years, the intravenous infection of rhesus macaques with SHIV-KS661 has consistently resulted in high viremia and CD4^+ ^T cell depletion, followed by malignant morbidity as a result of severe chronic diarrhea and wasting after 6 to 18 months [8]. Despite this well-developed *in vivo *model, the detailed kinetics of SHIV-KS661 remain unclear. Quantifying and understanding viral kinetics will provide us with novel insights about the pathogenesis of SHIV (and HIV-1), for example, by enabling the quantitative comparison of the replicative capacity of different strains.

In recent years, virological data from clinical patient studies, animal experiments, and cell culture studies have frequently been analyzed using mathematical models. Mathematical analysis of clinical data is an increasingly popular tool for the evaluation of drugs, the elaboration of diagnostic criteria, and the generation of recommendations for effective therapies [9-17]. Analyses of animal and cell culture studies have revealed fundamental aspects of viral infections including the specification of the half-life of infected cells and virus, the virus burst size, and the relative contribution of the immune response [18-29]. Important results have also been obtained in the analysis of purely *in vitro *experiments. For example, in Beauchemin *et. al*. [19], simple mathematical models were employed to analyze the effect of amantadine treatment on the course of experimental infections of Madin Darby canine kidney (MDCK) cells with influenza A/Albany/1/98 (H3N2) in a hollow-fiber (HF) reactor. Fits of the models to the experimental data determined that the 50% inhibitory concentration (IC_50_) of amantadine for that particular strain was 0.3-0.4 μM and found amantadine to be 56-74% effective at blocking the infection of target cells. Thus, analyses of experimental data using mathematical models have provided, and continue to provide, quantitative information about the kinetics of viral infections - particularly for HIV-1, the hepatitis C virus (HCV), and the influenza virus - by estimating infection parameters buried within experimental data.

Despite these successes, the available virological data, even for *in vitro *experiments, have often been limited in that many modeling analyses have been based only on total viral load data (e.g., RNA or DNA copies, hemagglutination assay (HA)) [9-13,15-17,20,22,23,26,27] or infectious viral load data (e.g., 50% tissue culture infection dose (TCID_50_) or plaque forming units (PFU)) [18,19,25]. Thus, while the applied mathematical models typically depend on the interaction of many components of the infection - including the populations of susceptible and infected cells - they are often only confronted by a single biological quantity: the time-course of the viral load. More rarely, diverse data sets including both virus and cell measurements have been considered [14,29-37]. Notable examples of the latter case include the analysis of an influenza infection in a microcarrier culture by Schulz-Horsel *et al*. [29], who measured and modeled the infectious and total viral load, along with the fraction of infected cells; and the *in vivo *studies of HIV-1 dynamics following antiviral therapy by Perelson and co-workers (e.g., [14,31]), who have considered measurements of viral load as well as susceptible and infected cells.

Here, we combined a relatively simple mathematical model of SHIV infection in HSC-F cells with an *in vitro *experimental system which allows for the measurement of both total and infectious viral load and the concentration of target and infected cells. We infected HSC-F - a CD4^+ ^T cell line established from cynomolgus monkey - *in vitro *with SHIV-KS661 at four different multiplicities of infection (MOI) and measured the concentration of Nef-negative (susceptible/target) and Nef-positive (infected/virus producing) HSC-F cells [cells/ml], and the total [RNA copies/ml] and infectious [TCID_50_/ml] viral load daily over nine days. With this abundant and diverse data, we were able to fully parameterize the dynamic model and determine robust estimates for viral kinetics parameters, thus quantifying the infection cycle. Our *in vitro *quantification system for SHIV-KS661 should be a valuable complement to the well-developed *in vivo *model and can be used to significantly improve the understanding of SHIV and HIV-1 pathogenesis.

## Results

### Mathematical model

To describe the *in vitro *kinetics of the SHIV-KS661 viral infection in our experimental system (Table 1), we expanded a basic mathematical model widely used for analyzing viral kinetics [13,17-19,27,38,39]. The following equations are our extended model:

**Table 1:** Experimental data for the *in vitro *experiment

MOI	Measurement day								
	
	0	1	2	3	4	5	6	7	8
Concentration of Nef-negative HSC-F cells (cells/ml)									

2 × 10^-3^	5470829	6044623	2690861	1012828	223584	42130	58470	10386	10270
2 × 10^-4^	2333804	4953074	2985268	2201172	811240	621750	60255	19998	4857
2 × 10^-5^	2574201	3563431	3434160	2345412	1269216	1345728	264794	71792	127996
2 × 10^-6^	3357117	2583058	4557411	35074989	1334060	1896048	1022157	307908	153360

Concentration of Nef-positive HSC-F cells (cells/ml)									

2 × 10^-3^	d.l.*	d.l.	439139	1167172	736416	177870	41530	19614	9730
2 × 10^-4^	d.l.	d.l.	84732	158828	548760	878250	89745	40002	5143
2 × 10^-5^	d.l.	d.l.	d.l.	64588	170784	574272	165206	88208	92004
2 × 10^-6^	d.l.	d.l.	d.l.	d.l.	65940	383952	347843	232092	86640

Total viral load of SHIV-KS661 (RNA copies/ml)									

2 × 10^-3^	9180000	331000000	2840000000	4050000000	3140000000	1120000000	154000000	20200000	5650000
2 × 10^-4^	1030000	26200000	256000000	1670000000	2110000000	1740000000	609000000	134000000	19400000
2 × 10^-5^	126744	4370000	51200000	489000000	1280000000	1940000000	1230000000	570000000	130000000
2 × 10^-6^	10170	800536	4600000	54200000	322000000	1300000000	1210000000	603000000	275000000

Infectious viral load of SHIV-KS661 (TCID_50_/ml)									

2 × 10^-3^	40	4064	40960	81920	163840	20480	2560	160	d.l.
2 × 10^-4^	d.l.	101	403	5120	16255	40960	1280	101	40
2 × 10^-5^	d.l.	64	640	4064	20480	25803	5120	1280	640
2 × 10^-6^	40	40	80	640	5120	1280	1280	640	1280

**Figure 1:**
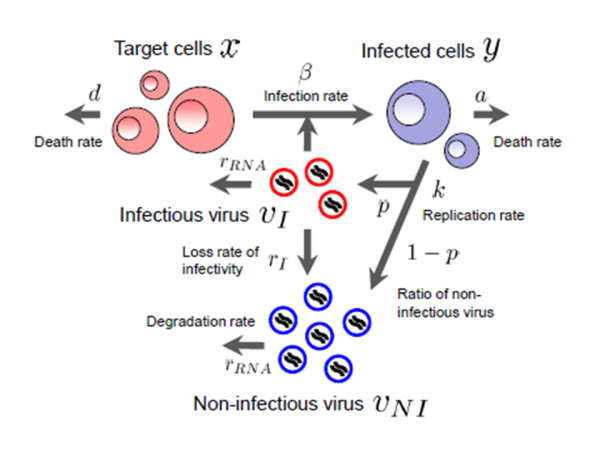
**A schematic representation of the mathematical model**. The variables *x *and *y *are the number of target and infected cells; *v_I _*and *v_NI _*are the number of RNA copies of infectious and non-infectious virus, respectively. Parameters *d*, *a*, *r_RNA_*, and *β *represent the death rate of target cells, the death rate of infected cells, the degradation rate of viral RNA, and the rate constant for infection of cells by infectious virus, respectively. It is assumed that infected cells release virus particles at a rate *k*, that a fraction *p *of these particles are infectious (*1-p *are non-infectious) but lose infectivity at a rate *r_I_*, becoming non-infectious.

"d.l." designates samples in which the concentration was below the detection limit.

(1) $$\frac{dx}{dt}=-\beta xv_{I}-dx$$

(2) $$\frac{dy}{dt}=\beta xv_{I}-ay$$

(3) $$\frac{dv_{I}}{dt}=pky-r_{I}v_{I}-r_{RNA}v_{I}$$

(4) $$\frac{dv_{NI}}{dt}=\left(1-p\right)ky+r_{I}v_{I}-r_{RNA}v_{NI}$$

where *x *and *y *are the number of target (susceptible) and infected (virus-producing) cells per ml of medium, *v_I _*and *v_NI _*are the number of RNA copies of infectious and non-infectious virus per ml of medium, respectively. Parameters *d*, *a*, *r_RNA_*, and *β *represent the death rate of target cells, the death rate of infected cells, the degradation rate of viral RNA, and the rate constant for infection of target cells by virus, respectively. We assume that each infected cell releases *k *virus particles per day (i.e., *k *is the viral production rate of an infected cell), of which a fraction *p *are infectious and *1-p *are non-infectious. Infectious virions lose infectivity at rate *r_I_*, becoming non-infectious. Implicit in Eqs.(1)-(4) is the assumption that once a cell is infected by infectious virus it immediately begins producing progeny virus. We also tested a variant of the model which incorporates an "eclipse" phase of infection to represent the cell's period of latency prior to virus production. We found, however, that including this phase did not significantly improve the fit of the model to the data and led to very similar extracted parameter values (see Additional files 1, 2, 3). Therefore, in all further analyses, this phase was omitted in favor of the simpler model formulation. A schematic of our mathematical model is shown in Figure 1.

To fit the observed viral load data - consisting of RNA copies/ml and TCID_50_/ml - and to account for the partial removal of cells and virus due to sampling, we transformed Eqs.(1)-(4) into the following scaled model:

(5) $$\frac{dx}{dt}=-\beta_{50}xv_{50}-dx-\delta x$$

(6) $$\frac{dy}{dt}=\beta_{50}xv_{50}-ay-\delta y$$

(7) $$\frac{dv_{RNA}}{dt}=ky-r_{RNA}v_{RNA}-r_{c}v_{RNA}$$

(8) $$\frac{dv_{50}}{dt}=k_{50}y-r_{I}v_{50}-r_{RNA}v_{50}-r_{c}v_{50}$$

where *v_RNA _= v_I_+v_NI _*is the total concentration of viral RNA copies, *v_50 _= αv_I _*is the infectious viral load expressed in TCID_50_/ml, and *α *is the conversion factor from infectious viral RNA copies to TCID_50_. Since the measure of 1 TCID_50 _corresponds to an average of 0.68 infection events (by Poisson statistics), we have *0 *<*α*≦*1.47 *TCID_50 _per RNA copies of infectious virus. Parameters *β_50 _= β/α *and *k_50 _= αpk *are the converted infection rate constant and production rate of infectious virus, respectively. At each sampling time, the concentration of Nef-negative and Nef-positive HSC-F cells must be reduced in our model by 5.5% and the viral loads (RNA copies and TCID_50_) by 99.93% to account for the experimental harvesting of cells and virus. These losses were modeled in Eqs.(5)-(8) by approximating the sampling of cells and virus as a continuous exponential decay, yielding a rate of *δ = 0.057 *per day for cell harvest and *r_c _= 7.31 *per day for virus harvest. We found that a model which implements the sampling explicitly, as a punctual reduction at each sampling time, similar to the model in [19], did not significantly improve the quality of the fit (data not shown).

Of the seven free model parameters remaining, three of them (*d*, *r_I_*, *r_RNA_*) were determined by direct measurements in separate experiments described below. The remaining four parameters (*β_50_*, *a*, *k*, *k_50_*) along with 16 initial (*t = 0*) values for the variables (four at each of the four MOI values) were determined by fitting the model to the data as described in **Methods **(Tables 2 and 3).

**Table 2:** Parameters values and derived quantities for the *in vitro *experiment

Parameter Name	Symbol	Unit	Value	95%CI
Calculated parameters for the continuous approximation of cell and virus harvest				

Harvest rate of target and infected cells	*δ*	day^-1^	0.057	--
Harvest rate of total and infectious virus	*r_c_*	day^-1^	7.31	--

Fitted parameters from separate experiments				

Decay rate of uninfected cells	*d*	day^-1^	0.21	0.17-0.26
Rate of virion infectivity loss	*r_I_*	day^-1^	0.93	0.44-1.4
Degradation rate of virion RNA	*r_RNA_*	day^-1^	0.039	0.013-0.065

Parameters obtained from simultaneous fit to full *in vitro *dataset				

Rate constant for infections	*β_50_*	(TCID_50_/ml·day)^-1^	4.95 × 10^-5^	(2.35-9.59) × 10^-5^
Decay rate of infected cells	*a*	day^-1^	1.18	0.85-1.26
Production rate of total virus	*k*	RNA copies·day^-1^	2.61 × 10^4^	(1.55-3.70) × 10^4^
Production rate of infectious virus	*k_50_*	TCID_50_·day^-1^	0.22	0.12-0.40

Quantities derived from fitted values				

Viral burst size (total)	*k/a*	RNA copies	2.21 × 10^4^	(1.74-2.96) × 10^4^
Viral burst size (infectious)	*k_50_/a*	TCID_50_	0.19	0.11-0.33
Basic reproductive number (without removal)	*R_0_*	--	62.8	51.1-76.8
Basic reproductive number (with removal)	R_0_*	--	7.01	5.70-8.45
Minimum fraction of infectious virus	*k_50_/k*	TCID_50_/RNA copies	8.63 × 10^-6^	(4.53-16.9) × 10^-6^

**Table 3:** Fitted initial (t = 0) values for the *in vitro *experiment

Variable	Unit	Fitted initial value at MOI of			
		
		2 × 10^-3^	2 × 10^-4^	2 × 10^-5^	2 × 10^-6^
*x_j_(0)*	cells/ml	6.55 × 10^6^	6.50 × 10^6^	5.82 × 10^6^	4.94 × 10^6^
*y_j_(0)*	cells/ml	6.47 × 10^2^	1.60 × 10^2^	6.89 × 10^-3^	0.254
*v_RNAj_(0)*	RNA copies/ml	9.15 × 10^6^	1.05 × 10^6^	1.58 × 10^5^	8.21 × 10^3^
*v_50j_(0)*	TCID_50_/ml	43.1	0.162	2.92	2.99

### *In vitro *half-lives of the SHIV-KS661 virus and HSC-F cells

**Figure 2:**
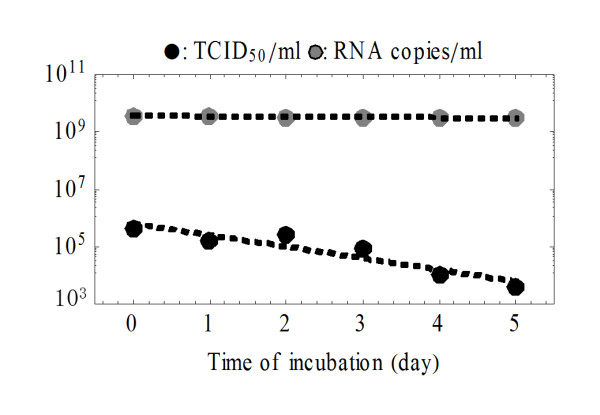
**Rates of RNA degradation and loss of infectivity for SHIV-KS661**. Stock virus was incubated under the same conditions as the infection experiments, but in the absence of cells, then sampled every day and stored at -80°C. After the sampling, the RNA copy number (gray circles) and 50% tissue culture infectious dose (black circles) of the samples were measured. Linear regressions yielded a rate of RNA degradation of *r_RNA _= 0.039 *per day and a rate of loss of viral infectivity of *r_I _= 0.93 *per day.

The rates at which SHIV-KS661 virions lose infectivity, *r_I_*, and the rate at which their viral RNA degrades, *r_RNA_*, were each estimated directly in separate experiments (Figure 2). Linear regressions were performed to fit *logv_RNA_(t) = logv_RNA_(0)-r_RNA_t *and *logv_50_(t) = logv_50_(0)-r_I_t *to those data, yielding values of *r_RNA _= 0.039 *per day (95% confidence interval (95%CI): *0.013-0.065 *per day) and *r_I _= 0.93 *per day (95%CI: *0.44-1.4 *per day). These correspond to an infectious virion half-life of *17.9 *h and an RNA viability half-life of *17.7 *d. The death rate of target cells, *d*, was also estimated directly, in a mock infection experiment where Nef-negative (target) HSC-F cells were exposed to the culture conditions of the experiment without virus (data not shown). A linear regression was performed to fit *logx(t) = logx(0)-(d+δ)t *to the time course data, yielding *d = 0.21 *per day (95%CI: *0.18-0.27*), corresponding to an average target cell lifespan of *4.76 *d (half-life of *3.30 *d).

### Time-course *in vitro *data

**Figure 3:**
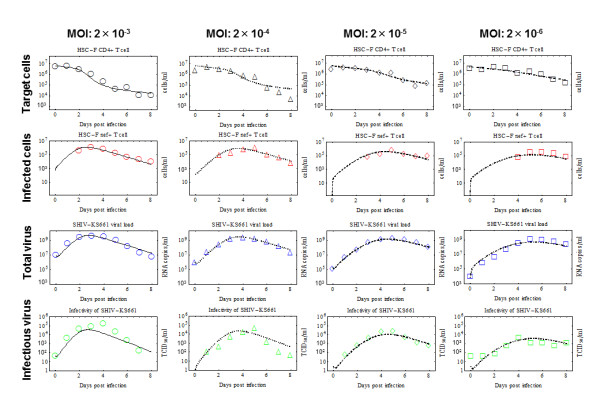
**Fits of the mathematical model to experimental data of SHIV-KS661 *in vitro***. HSC-F cells were inoculated with SHIV-KS661 24 h before *t = 0*, and each *in vitro *experimental quantity was measured daily from *t = 0 *d to *8 *d. The curves show the best-fit of the model (Eqs.(5)-(8), lines) to the experimental data (points) for the target cells, infected cells, and the total and infectious viral load for the four different experiments conducted at different MOIs. All data were fitted simultaneously as described in the text. The fitted *t = 0 *values of each quantity are given in **Table 3**.

Time-course *in vitro *experimental data were collected over nine days, consisting of the concentrations of Nef-negative and Nef-positive HSC-F cells [cells/ml], the total SHIV-KS661 viral load [RNA copies/ml], and the infectious viral load [TCID_50_/ml]. At each daily measurement, almost all of the culture supernatant (99.93%) was removed for viral counting; a small percentage of cells (5.5%) were removed for counting and FACS analysis, and the remaining cells were thoroughly washed and replaced in fresh medium. The experiment was repeated for four different values of the initial viral inoculum (MOI). In total, we obtained 130 data points for quantifying SHIV-KS661 viral kinetics *in vitro *(Table 1** and **Figure 3).

In examining the MOI = *2 × 10^-3 ^*data, one can see that the target cell population remains high (near its initial value of approximately *6.46 × 10^6 ^*cells/ml) until just before the peak of the virus concentration, at which point the target cell population decreases rapidly. The total infected cell population, the total virus count (RNA/ml), and the infectious virus count (TCID_50_/ml) all peak around *t = 3 *days. Moreover, the rate of exponential decay (downward slope) of the total virus and the infected cell population after their respective peaks are quite similar. This behavior is expected: since the virus is being almost completely removed from the culture on a daily basis due to sampling and the RNA degradation rate is very small (*r_RNA _= 0.039 *per day); the measured RNA count of virus is nearly equal to the total number of virus produced over the preceding day which should be proportional to the number of cells producing virus. Similar reasoning should apply to the decay of infectious virus - the net infectious virus measured after one day should also be approximately proportional to the number of infected cells - but the rates appear much less closely aligned in this case, perhaps due to larger errors in the TCID_50 _measurement technique. Alternatively, the observed more rapid than expected decrease of infectious virus could have a biological cause. For instance, the co-infection of cells by competent and defective interfering viruses at late stages in the experiment could lead to an enhanced production of the latter [40], thus successively reducing the fraction of infectious particles. An increase in cell-death by-products could also contribute to the decline in virus infectivity. In SIV and SHIV infections *in vivo*, a decreasing viral infectiousness has been observed over time [7,41,42], but the timescale of this decay is longer than that observed here and likely has an in-host origin.

A comparison of the experiments at the four different MOI values shows that a decrease in the initial viral inoculum serves primarily to delay the course of the infection. The target cell populations drop to approximately half of their original values at *t ≈ 1.9*, *2.6*, *3.5 *and *4.1 *days, respectively, for the four experiments in order of decreasing MOI. Similarly the peaks of the total viral RNA occur at *t ≈ 3.0*, *4.0*, *5.0 *and *5.5 *days, respectively. The experiments at lower MOI have slightly lower viral and infected cell peaks, but differ from those of the experiment at MOI = *2 × 10^-3 ^*by less than a factor of three.

### Relevant SHIV-KS661 viral kinetics measures

Having fixed the values of the rates of virion decay (*r_I _*and *r_RNA_*) and the target cell death rate (*d*) using separate experiments, we estimated the values and 95% CI of the four remaining unknown parameters (*β_50_*, *a*, *k*, *k_50_*) by fitting the model in Eqs.(5)-(8) to the full *in vitro *dataset simultaneously (Table 2). The death rate of infected cells was determined to be *a = 1.18 *per day (95%CI: *0.85-1.26 *per day) which implies that the half-life of infected cells (i.e., *log2/a*) is *14.1 *h. Infected HSC-F cells were found to produce *k = 2.61 × 10^4 ^*RNA copies of virus per day.

From the directly fitted parameters, we also calculated a number of important derived quantities and their 95% CI, determined from the bootstrap fits (Table 2). One key measurement of viral kinetics is the viral burst size, which is the total number of virus produced by an infected cell during its lifetime [18-20]. The total burst size of SHIV-KS661 (including non-infectious and infectious virus) is given in our model by *k/a *and was estimated from our *in vitro *experiment to be *2.21 × 10^4 ^*RNA copies. The burst size of infectious SHIV-KS661, *k_50_/a*, was *0.19 *TCID_50_.

To broadly characterize viral kinetics, it is instructive to calculate the basic reproductive number for the system, which has the form *R_0 _= β_50_k_50_x_0_/(a(r_I_+r_RNA_)) *and is interpreted as the number of newly infected cells intrinsically generated by a single infectious cell at the start of the infection [15-19,27]. The initial number of HSC-F cells, *x_0_*, was approximately *6.46 × 10^6 ^*cells/ml, which, together with the values of the five estimated parameters, yields an estimate for the basic reproductive number of *62.8*. This large value (6*2.8»1*) implies that, given a small initiating infected cell population, the infection is overwhelmingly likely to spread to the entire population of cells.

After the repetitive removal of cells and virus begins, the basic reproductive number is effectively reduced, much like the effect of quarantine on the epidemiological measure of *R_0_*. When the effects of removal are included in the calculation of the basic reproductive number it has the form R_0_* = β_50_k_50_x_0_/((a+δ)(r_I_+r_RNA_+r_C_)) which yields a smaller value of 7.01. This value better characterizes the course of the infection in our system, for example, through the recursive relation for the approximate fraction of eventually infected cells, f_I _= 1- exp(-R_0_* f_I_) [43]. Using this expression, we find that the fraction of target cells at the end of the infection (*1-f_I_*) should be *9.01 × 10^-4^*, which implies an approximately final target cell concentration is *5.87 × 10^3 ^*cells/ml. This value agrees well with the asymptotic concentration of Nef-negative HSC-F cells in the MOI = *2 × 10^-3 ^*experiment, ~*1.03 × 10^4 ^*cells/ml. The delay of the infection precludes an estimate of the final target cell value at smaller MOI values.

Our model formulation also enables us to determine, albiet not fully, two interesting quantities related to the infectiousness of SHIV-KS66 virions. Parameter *p *(where *0 *<*p*≦*1*) is the fraction of SHIV-KS66 virions which are infectious at the time of production: the larger the value of *p*, the fewer defective virus particles are produced by infectious cells. Parameter α is approximately the fraction of infectious virions which are measured in the TCID_50 _assay, i.e., it is the ratio of TCID_50 _viral titer (*v_50_*) to the RNA count of infectious virions (*v_I_*). It follows from Poisson statistics that *0 *<*α*≦*1.47 *TCID_50 _per infectious RNA copies of infectious virions. While we cannot determine *p *and *α *individually in our analysis, their product is given by *k_50_/k = (αpk)/k = αp = 8.63 × 10^-6 ^*TCID_50 _per infectious RNA copies. Because of the upper bounds on *p *and *α*, the value of their product imposes a minimum condition on each: *5.87 × 10^-6 ^< p ≦ 1 *and *8.63 × 10^-6 ^*<*α*≦*1.47 *TCID_50 _per RNA copies.

We can constrain these parameters further by considering the basic reproductive number *R_0 _= 62.8*, which implies that one infectious cell will infect *62.8 *other cells over the course of its infectious lifespan. Thus, one infectious cell must produce at least *62.8 *infectious virions over its lifespan, i.e., have a burst size of at least *62.8 *infectious RNA copies. The burst size in infectious virions is given by *pk/a*, so this requirement can be written as *pk/a ≥ R_0 _*infectious RNA copies (or, equivalently, *p ≥ aR_0_/k *infectious RNA copies) which, based on the values of these quantities from Table 2 implies that *p ≥ 2.84 × 10^-3^*. Thus *2.84 × 10^-3^*≦*p*≦*1*, which means that at least one in every 350 virions produced is infectious. Since *αp = 8.63 × 10^-6 ^*TCID_50 _per infectious RNA copies, it follows that *8.63 × 10^-6 ^*<*α*≦*3.04 × 10^-3 ^*TCID_50 _per infectious RNA copies, which means that 1 TCID_50 _corresponds to at least *330 *(*1/3.04 × 10^-3^*) infectious virus, but perhaps as many as *120, 000 *(*1/8.63 × 10^-6^*).

## Discussion

We have applied a simple mathematical model to quantitatively characterize the *in vitro *kinetics of SHIV-KS661 virus infection in HSC-F cell cultures, leveraging experimental data for total and infectious viral load, along with target and infected cell dynamics, to fully parameterize the system. Specifically, we determined values for the rate of loss of infectivity and the RNA degradation rate of SHIV-KS661, the target and infected HSC-F cell half-life, the rate constant for infection of target cells and the infectious and total viral production rates of infected cells. From these fundamental quantities, we also estimated a number of important derived quantities, including the burst size of an infected cell and the basic reproductive number. Additionally, by measuring both the total and infectious viral load within the context of a mathematical model we were able to provide a lower bound for the proportion of infectious virions produced by infected cells.

We estimated the half-life of SHIV-infected HSC-F cells to be *14.1 *h. In clinical studies of patients or animals, it is extremely difficult to continuously measure the number of infected cells during infection. This is because the amount of infected cells in peripheral blood (PB) is very small. For example, in HIV-1 infected patients, there are only about 10^2 ^infected cells per 10^6 ^peripheral blood mononuclear cells at their set point [14]. Thus, measuring the number of infected cells in PB during the early phase of infection is technically difficult. In HIV-1 humanized mice, infected cells in PB are not detectable even during the acute phase when 80-90% of target cells in the spleen and lymph nodes are infected (K. Sato and S. Iwami, unpublished data). For this reason, the death rate of infected cells *in vivo *has primarily been estimated from the viral load decay (or the decay of infectious virus) after the peak of an acute infection [11,16,17,20,27] or after antiviral drug administration [10,14,15,22]. The maximum half-lives of HIV-1 and SIV-infected cells were both initially estimated - by analysis of *in vivo *viral decay under antiviral therapy - to be ~24 h [14,27], but drug combinations with higher efficacy have reduced the estimates to ~17 and ~11 h, respectively [12,22,44]. Our *in vitro *estimate of the half-life, based on direct observations of Nef-positive cell decay, agrees well with these indirect *in vivo *measures, despite the absence of immune effects.

We determined an SHIV-KS661 viral burst size of *2.21 × 10^4 ^*RNA or *0.19 *TCID_50 _for HSC-F cells. Current estimates of viral burst size in the literature rely on inhibiting multiple rounds of infection by antiviral drugs, washouts of infected cells, serial dilutions of infected cells, or infection by single-cycle virus [11,20,21,45,46]. The inhibition of the multiple rounds of infection, however, can introduce additional confounding factors on the viral burst size as discussed in [20]. Here, we have calculated the burst size of SHIV-KS661 in HSC-F cells indirectly by estimating the viral production rate and the average lifespan of infected cells over the course of a typical infection. Our estimate is quite close to the ~5 × 10^4 ^RNA value determined in recent SIV single-cycle virion experiments *in vivo *[20], which, notably, was 10-100 times higher than most previously measured values. We also calculated a basic reproductive number for SHIV-KS661 in HSC-F cell cultures as approximately *62.8 *for the initial stages of the infection and approximately *7.01 *for the entire course, when the effects of manual removal of virus and cells are included. The latter value implies that reducing viral growth by about 85.7% for the entire course with antiviral intervention, for example, would prevent viral spread *in vitro *given the daily sampling.

It is widely believed that retroviruses are predominantly defective, with less than 0.1% of virions in plasma or culture media being infectious [47-49]. On the other hand, it has recently been suggested that HIV-1 virions, for example, are inherently highly infectious, but that slow viral diffusion in liquid media and rapid dissociation of virions from cells severely limit infections in cultures (i.e., in assays measuring infectivity) [50,51]. On both sides of this debate, however, studies have often relied on measurements of the proportion of infectious virus in stock samples, or on measurements of the infectious/non-infectious ratio over the course of an *in vitro *experiment. These direct measurements of the infectivity ratio in a virus sample are necessarily confounded by a continuous loss of infectious virus, driven by thermal deactivation and RNA degradation and, as such, these analyses cannot address the question of what fraction of virus are infectious at the time of production. Here, we have estimated the production rates of both infectious and non-infectious virus, allowing for a novel quantitative specification of the fraction of newly generated virus that is infectious. This fundamental quantity is important in understanding the role and influence of defective virus particles [48-50,52]; and, to our knowledge, this has not been measured before for any virus strain. We determined the theoretical minimum value for the proportion of infectious virions among newly produced virus, *p*, to be *8.62 × 10^-6^*, by calculating the ratio of the infectious to total viral production rates *k_50_/k*. The ratio of the production rates, however, is actually *p *multiplied by *α*, where *α *is the conversion factor from RNA count of infectious virions to TCID_50 _(i.e., roughly the fraction of infectious virions that are actually measured in a TCID_50 _titration assay). Therefore, since *α *is likely much less than one, the proportion of infectious virus is likely much higher. In fact, using the measured basic reproductive number, we estimate that the minimum value of *p *is approximately *2.84 × 10^-3^*, meaning that at least 1 of every 350 virions produced is infectious. Determining this quantity is particularly important in determining the true efficiency of infectious virus replication. In previous publications [53,54], it was reported that *vif*-deficient HIV-1 showed decreased production of infectious virus due to the inhibition of the viral replication process by host factors such as APOBEC3 protein. Our method suggests a novel and more reliable way to determine the effect of the host-viral protein interaction on infectious viral replication.

In another aspect of viral infectivity, we found that the SHIV-KS661 virion infectious half-life at 37°C was 17.9 h. While this quantity is vital for understanding viral dynamics *in vitro*, and represents an important, strain-specific physical property of the virion, it is unlikely to strongly influence *in vivo *dynamics, due to the extremely high physical clearance rate in the blood (virion half-lives are on the order of minutes) [23].

## Conclusions

To conclude, by using a simple mathematical model for SHIV-KS661 infection on HSC-F cells and an abundant, diverse experimental dataset, we have been able to reliably estimate the parameters characterizing cell-virus interactions *in vitro*. Based on these estimated parameters, we have provided a quantitative description of SHIV-KS661 kinetics in HSC-F cell cultures which is consistent with previous studies of lentiviruses and provides a number of novel quantities. Most notably, our analysis provides an estimate of the minimum fraction of infectious virus produced by an infected cell. Our improved method for quantifying viral kinetics *in vitro *- which depends crucially on detailed time-course information about the infection of cells in addition to that of virus (both total particle count and infectious titer) - could be applied to other viral infections. The method could likely improve the understanding of the differences in replication across different strains [25,55] or between complete and protein-deficient viruses [53,54]; the differences in viral pathogenesis [6]; and the effects of anti-viral therapies [9,13]. Quantifying the *in vitro *viral kinetics for viruses such as HCV [56,57], for which a convenient animal experimental model has not been established, is of particular interest. Since the method presented here allows for the complete resolution of all viral kinetic parameters, it also enables the identification of the mechanisms of action for new antiviral compounds. Indeed, repeating the experimental infection under various antiviral concentrations would distinctly reveal which parameters (e.g., half-life of infected cells, infectious viral burst size) are affected by the antiviral and to what extent. Furthermore, the inhibitory concentration of the compound could be independently determined for each parameter. Thus, our synergistic approach, combining experiments and mathematical models, has broad potential applications in virology.

## Methods

### Virus and cell culture

The virus stock of SHIV-KS661 [5] was prepared in a CD4^+ ^human T lymphoid cell line, M8166 (a subclone of C8166) [58]. The stock was stored in liquid nitrogen until use. Establishment of the HSC-F cell line has been previously described [59]. This is a cynomolgous monkey CD4^+ ^T-cell line from fetal splenocytes that were immortalized by infection with Herpesvirus saimiri subtype C. The cells were cultured in RPMI-1640 medium supplemented with 10% fetal calf serum at 37°C and 5% CO_2 _in humidified condition.

### *In vitro *experiment

Each experiment was performed using 2 wells of a 24-well plate with a total suspension volume of 2 ml (1 ml per well) and an initial cell concentration of *6.46 × 10^6 ^*cells/ml in each well. Because the initial cell concentration is close to the carrying capacity of 24-well plates and the doubling time of HSC-F cells is not short, the population of target cells, in the absence of SHIV-KS661 infection, changes very little on the timescale of our experiment. We therefore neglected the effects of potential regeneration of HSC-F cells when constructing the mathematical model.

Cultures of HSC-F cells were inoculated at different MOIs (*2.0 × 10^-3^, 2.0 × 10^-4^, 2.0 × 10^-5^, 2.0 × 10^-6^*; MOI = TCID50/cell) of SHIV-KS661 and incubated for 4 h at 37°C. After inoculation, cells were washed three times to remove the infection medium and placed in fresh media. Subsequently, the culture supernatant was harvested daily for 9 d, along with a small fraction of the cells (5.5%) for counting of viable and infected cells. The remaining cells were then gently washed three times and placed in a fresh, virus-free, medium. Separate experiments (not shown) determined that free virus was not completely removed, but that virus concentration in the supernatant dropped to 0.066% of its value prior to this sampling and washing procedure. Harvested culture supernatants were frozen and stored at -80°C until they were assayed via RT-PCR and TCID_50 _titration, as described below.

### Count of viable and infected cells

**Figure 4:**
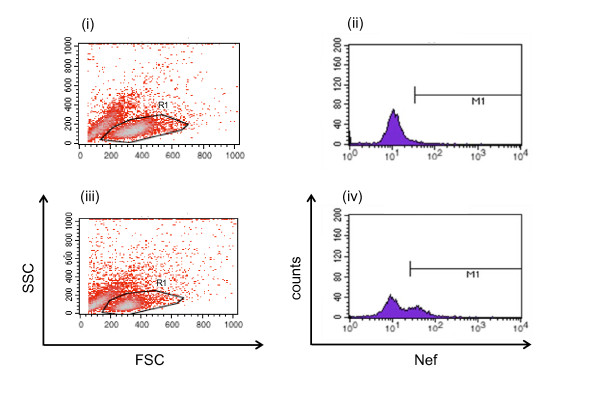
**Flow cytometry analysis of HSC-F cells stained with Nef antigen**. Representative data at 1 day (i and ii) and 5 days (iii and iv) post-inoculation with SHIV-KS661 at an MOI of 2 × 10^-5 ^are shown. The viable cell population, gated by the data of forward (FSC) and side scatter (SSC) (region surrounded with a solid line in (i) and (iii), respectively), was fractionated by Alexa-488-labeled Nef staining in (ii) and (iv). The counts within the M1 regions and the remaining parts of the total counts are defined as the fraction of Nef- positive (infected) and Nef-negative (target) cells, respectively.

Virus infection of the HSC-F cells was measured by FACS analysis using markers for intracellular SIV Nef antigen expression. The counts of total and viable cells were first determined using a cell counting chamber (Burker-turk, Erma, Tokyo, Japan) with trypan blue staining. Viable HSC-F cells (gated by forward- and side-scatter results) were examined by flow cytometry to measure the intracellular SIV Nef antigen expression (see Figure 4). Cells were permeabilized with detergent-containing buffer (Permeabilizing Solution 2, BD Biosciences, San Jose, CA). The permeabilized cells were stained with anti-SIV Nef monoclonal antibody (04-001, Santa Cruz Biotechnology, Santa Cruz, CA) labeled by Zenon Alexa Fluor 488 (Invitrogen, Carlsbad, CA), and analyzed on FACSCalibur (BD Biosciences, San Jose, CA).

### Total and infectious viral load quantification

We followed the kinetics of both the total and infectious SHIV-KS661 viral load. The total viral load was measured with a real-time PCR quantification assay, as described previously [5], with minor modifications. Briefly, total RNA was isolated from the culture supernatants (140 μl) of virus-infected HSC-F cells with a QIAamp Viral RNA Mini kit (QIAGEN, Hilden, Germany). RT reactions and PCR were performed by a QuantiTect probe RT-PCR Kit (QIAGEN, Hilden, Germany) using the following primers for the *gag *region; SIV2-696F (5'-GGA AAT TAC CCA GTA CAA CAA ATAGG-3') and SIV2-784R (5'-TCT ATC AAT TTT ACC CAGGCA TTT A-3'). A labeled probe, SIV2-731T (5'-Fam-TGTCCA CCT GCC ATT AAG CCC G-Tamra-3'), was used for detection of the PCR products. These reactions were performed with a Prism 7500 Sequence Detector (Applied Biosystems, Foster City, CA) and analyzed using the manufacturer's software. For each run, a standard curve was generated from dilutions whose copy numbers were known, and the RNA in the culture supernatant samples was quantified based on the standard curve. The infectious viral load was measured by TCID_50 _assay in HFC-S cell cultures using 96-well flat bottom plates at cell concentrations of 1.0 × 10^6 ^cells/ml. The titer of the virus was determined as described by Reed and Muench [60].

### Rate of RNA degradation and loss of infectivity for SHIV-KS661 in the culture condition

The RNA degradation and thermal deactivation of SHIV-KS661 was measured by incubating 4 ml of stock virus, without cells, in a 35 mm Petri dish under the same conditions as the infection experiments (in RPMI-1640 medium supplemented with 10% fetal calf serum at 37°C and 5% CO_2 _in humidified condition). Aliquots of the stock (500 μl) were sampled every day from day 0 to day 5 and stored at -80°C (see Figure 2). The RNA copy number and 50% tissue culture infectious dose of the samples were measured as described above.

### Mathematical model and fitting

We simultaneously fit Eqs.(5)-(8) to the concentration of Nef-negative and Nef-positive HSC-F cells and the infectious and total viral loads at four different MOIs (Figure 3) using nonlinear least-squares regression (FindMinimum package of *Mathematica7.0*) which minimizes the following objective function:

$$\begin{array}{c} SSR=\overset{4}{\underset{j=1}{\sum }}\left[\overset{9}{\underset{i=1}{\sum }}{\left\{\text{log}x_{j}\left(t_{i}\right)-\text{log}x_{j}^{e}\left(t_{i}\right)\right\}}^{2}+\right)\overset{9}{\underset{i=1}{\sum }}{\left\{\text{log}y_{j}\left(t_{i}\right)-\text{log}y_{j}^{e}\left(t_{i}\right)\right\}}^{2}\\ \left(+\overset{9}{\underset{i=1}{\sum }}{\left\{\text{log}{v_{RNA}}_{j}\left(t_{i}\right)-\text{log}{v_{RNA}}_{j}^{e}\left(t_{i}\right)\right\}}^{2}+\overset{9}{\underset{i=1}{\sum }}{\left\{\text{log}{v_{50}}_{j}\left(t_{i}\right)-\text{log}{v_{50}}_{j}^{e}\left(t_{i}\right)\right\}}^{2}\right] \end{array}$$

where *x_j_(t_i_), y_j_(t_i_), v_RNAj_(t_i_)*, and *v_50j_(t_i_) *are the model-predicted values for Nef-negative cells, Nef-positive cells, total RNA viral load and infectious (TCID_50_) viral load, given by the solution of Eqs.(5)-(8) at measurement time *t_i _*(*t_i _*= 0, 1, 2, ⋯, 8 d). Index *j *is a label for the MOI of the four experiments (i.e., for MOI: *2.0 × 10^-3^*, *2.0 × 10^-4^*, *2.0 × 10^-5^*, and *2.0 × 10^-6^*). The variables with superscript "*e*" are the corresponding experimental measurements of those quantities. Note that the HSC-F cells were inoculated with SHIV-KS661 24 h before *t = 0*. Experimental measurements below the detection limit (marked "d.l." in Table 1) were excluded when computing the *SSR*. Alternative fits with various weights on the infectious viral load to account for larger errors in the TCID_50 _value [61], were also performed, but these did not significantly alter the extracted parameter values (Additional files 4, 5, 6, 7, 8, 9). To derive the 95% confidence interval for each parameter, we employed the bootstrap method [62,63], estimating parameter values using 256 replicates of the four data sets and calculating the 2.5 and 97.5 percentiles.

## List of abbreviations

SHIV: simian/human immunodeficiency virus; HIV-1: human immunodeficiency virus type-1; MDCK: Madin Darby canine kidney; HF: hollow-fiber; IC_50_: 50% inhibitory concentration; HCV: hepatitis C virus; HA: hemagglutination assay; TCID_50_: 50% tissue culture infection dose; PFU: plaque forming units; MOI: multiplicities of infection; PB: peripheral blood.

## Competing interests

The authors declare that they have no competing interests.

## Authors' contributions

SI, KS, TI and TM designed the study.

SI, BPH and SM carried out data analysis.

TT and TM performed all experiments.

SI and CAAB developed mathematical model.

SI, BPH, CAAB and TM wrote the final manuscript.

All authors read and approved the final manuscript.

## Supplementary Material

Additional file 1 **Fit of a mathematical model which includes an eclipse phase of infection to experimental data of SHIV-KS661 *in vitro***. Testing a variant of the model which incorporates an "eclipse" phase of infection to represent the cell's period of latency prior to virus production (see Additional file 2 for more detailed information). Click here for file

Additional file 2 **Additional documentation for Additional files **1. Detailed explanation of mathematical models used in Additional files 1. Click here for file

Additional file 3 **Table for estimated parameters in Additional files **1. Parameters values, initial values and derived quantities for the *in vitro *experiment with eclipse model. Click here for file

Additional file 4 **Fit of the mathematical model with SSR^W ^(*W = 0.0001*) to experimental data of SHIV-KS661 *in vitro *(a)**. Fitting with weight of *W = 0.0001 *on the infectious viral load to account for larger errors in the TCID_50 _value (see Additional file 8 for more detailed information). Click here for file

Additional file 5 **Fit of the mathematical model with SSR^W ^(*W = 0.1*) to experimental data of SHIV-KS661 *in vitro *(b)**. Fitting with weight of *W = 0.1 *on the infectious viral load to account for larger errors in the TCID_50 _value (see Additional file 8 for more detailed information). Click here for file

Additional file 6 **Fit of the mathematical model with SSR^W ^(*W = 10*) to experimental data of SHIV-KS661 *in vitro *(c)**. Fitting with weight of *W = 10 *on the infectious viral load to account for larger errors in the TCID_50 _value (see Additional file 8 for more detailed information). Click here for file

Additional file 7 **Fit of the mathematical model with SSR^W ^(*W = 10000*) to experimental data of SHIV-KS661 *in vitro *(d)**. Fitting with weight of *W = 10000 *on the infectious viral load to account for larger errors in the TCID_50 _value (see Additional file 8 for more detailed information). Click here for file

Additional file 8 **Additional documentation for Additional files **4, 5, 6, 7. Detailed explanation of mathematical models used in **Additional files **4, 5, 6, 7. Click here for file

Additional file 9 **Table for estimated parameters in Additional files **4, 5, 6, 7. Parameters values and derived quantities for the *in vitro *experiment with various SSR^W^s. Click here for file
