# Supplementary figures and images for: Quantification system for the viral dynamics of a highly pathogenic simian/human immunodeficiency virus based on an in vitro experiment and a mathematical model
Source: Retrovirology. 2012 Feb 25;9:18. doi: 10.1186/1742-4690-9-18 (PMC3305505; doi:10.1186/1742-4690-9-18)

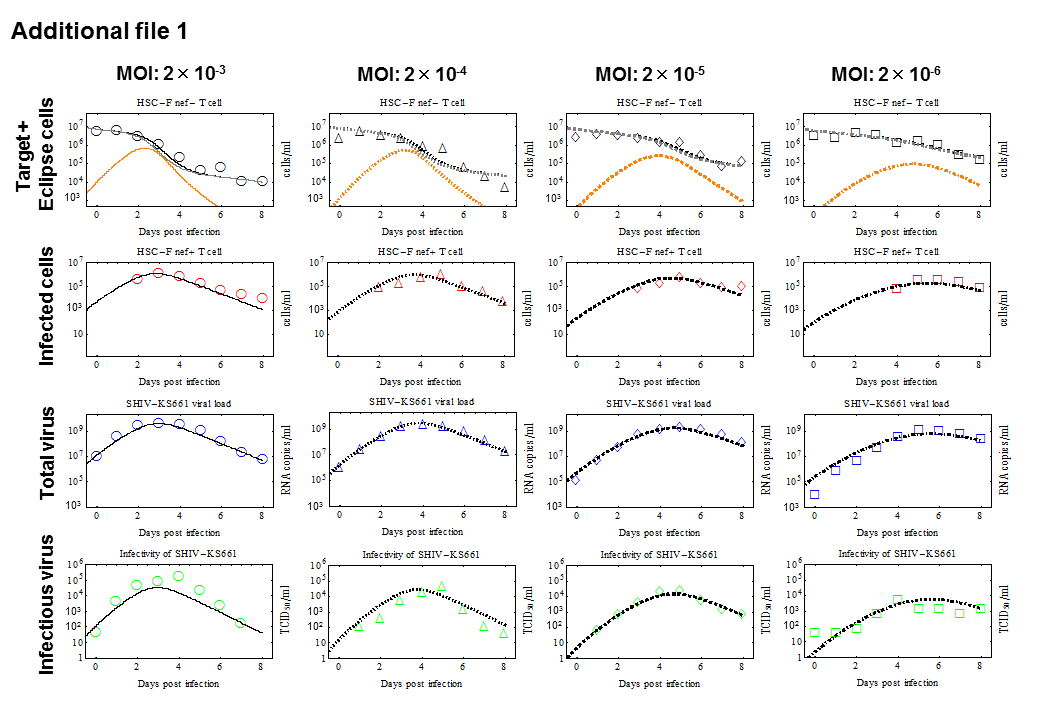

Supplement: Additional file 1 — Fit of a mathematical model which includes an eclipse phase of infection to experimental data of SHIV-KS661 in vitro. Testing a variant of the model which incorporates an "eclipse" phase of infection to represent the cell's period of latency prior to virus production (see Additional file 2 for more detailed information). [file 1742-4690-9-18-S1.TIFF]

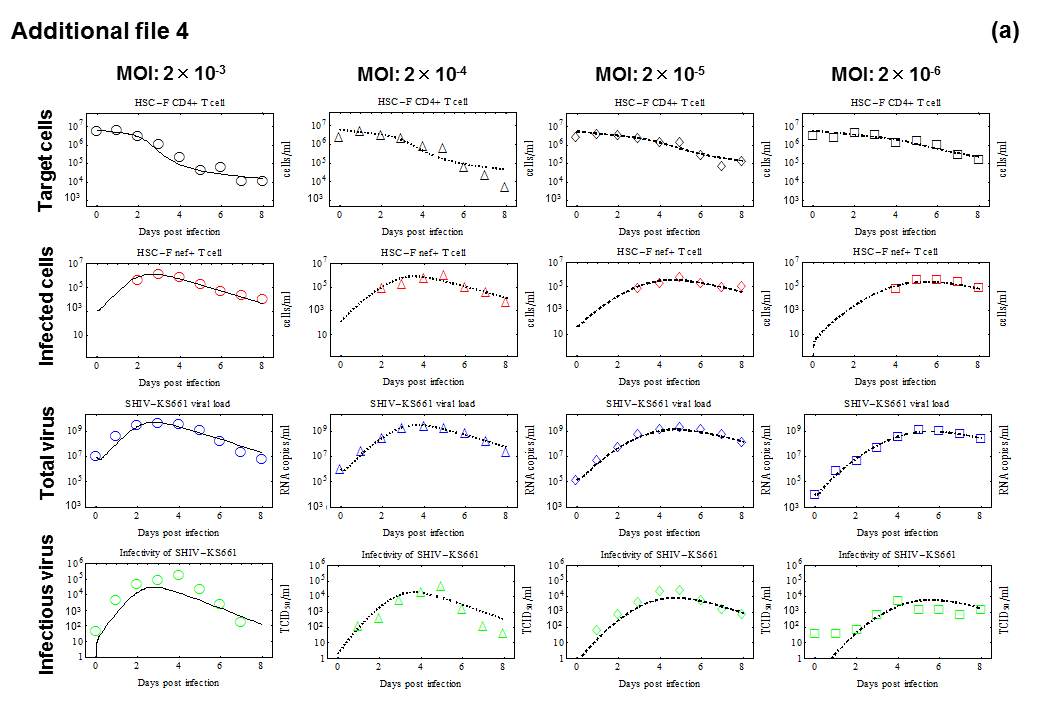

Supplement: Additional file 4 — Fit of the mathematical model with SSRW (W = 0.0001) to experimental data of SHIV-KS661 in vitro (a). Fitting with weight of W = 0.0001 on the infectious viral load to account for larger errors in the TCID50 value (see Additional file 8 for more detailed information). [file 1742-4690-9-18-S4.TIFF]

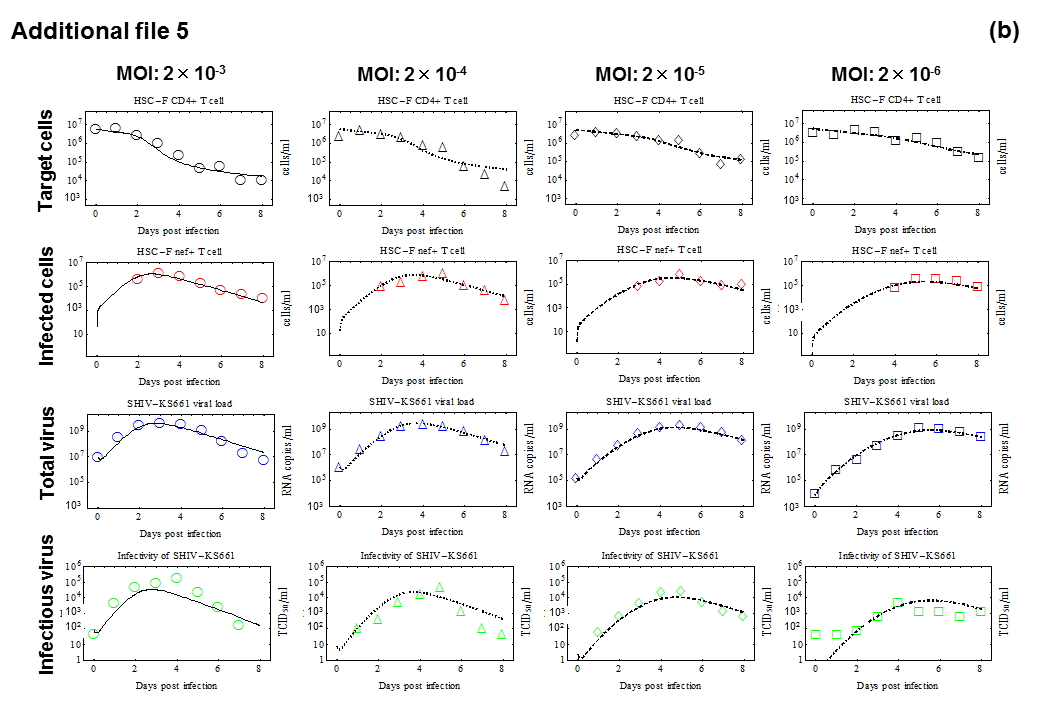

Supplement: Additional file 5 — Fit of the mathematical model with SSRW (W = 0.1) to experimental data of SHIV-KS661 in vitro (b). Fitting with weight of W = 0.1 on the infectious viral load to account for larger errors in the TCID50 value (see Additional file 8 for more detailed information). [file 1742-4690-9-18-S5.TIFF]

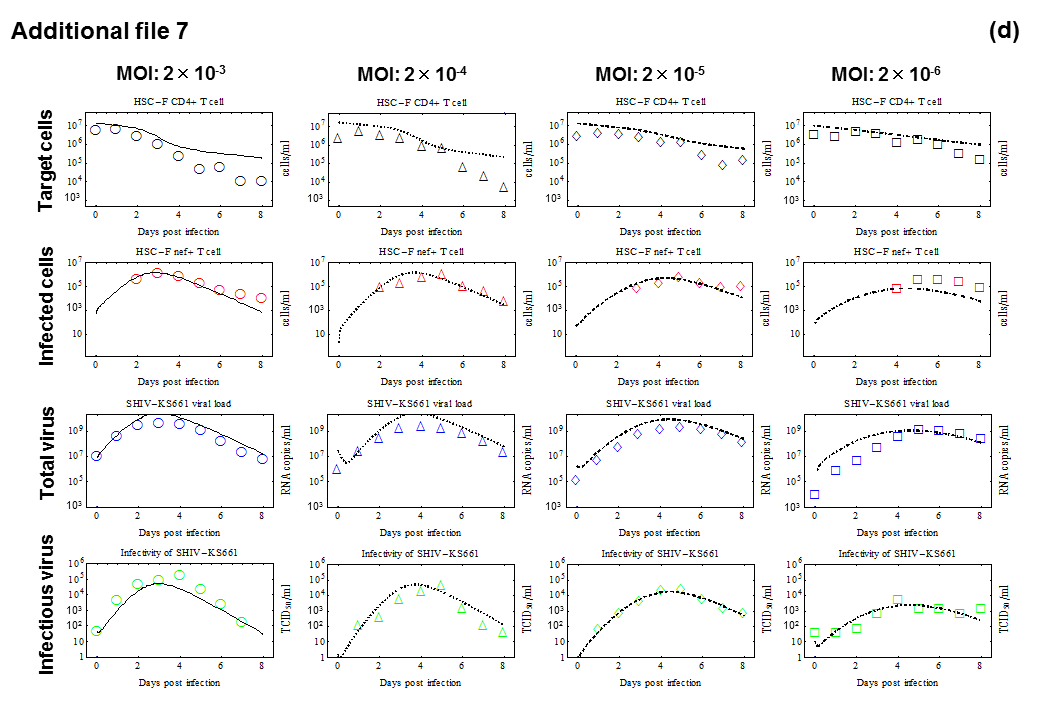

Supplement: Additional file 6 — Fit of the mathematical model with SSRW (W = 10) to experimental data of SHIV-KS661 in vitro (c). Fitting with weight of W = 10 on the infectious viral load to account for larger errors in the TCID50 value (see Additional file 8 for more detailed information). [file 1742-4690-9-18-S6.TIFF]
