# Supplementary material for: Quantification system for the viral dynamics of a highly pathogenic simian/human immunodeficiency virus based on an in vitro experiment and a mathematical model
Source: Retrovirology. 2012 Feb 25;9:18. doi: 10.1186/1742-4690-9-18 (PMC3305505; doi:10.1186/1742-4690-9-18)
Supplement: Additional file 2 — Additional documentation for Additional files 1. Detailed explanation of mathematical models used in Additional files 1. [file 1742-4690-9-18-S2.PDF]

EXPLANATION OF ADDITIONAL FILE 1.

Additional file 1. Fit of a mathematical model which includes an eclipse phase of infection to experimental data of SHIV-KS661 *in vitro*. To implement an eclipse phase we used the following delay differential equation (DDE) model:

$$dx/dt = -\beta x v_I - dx, dE/dt = \beta x v_I - \beta x(t-\tau_\varepsilon) v_I(t-\tau_\varepsilon), dy/dt$$
$$= \beta x(t-\tau_\varepsilon) v_I(t-\tau_\varepsilon) - ay, dv_I/dt = pky - r_I v_I - r_{RNA} v_I, dv_{NI}/dt$$
$$= (1-p)ky + r_I v_I - r_{RNA} v_{NI}$$

where E is the number of cells in the eclipse phase (infected, but not yet expressing Nef) and the parameter τ_ε represents the fixed delay prior to expression of Nef. Data were fitted using the same objective function used for the basic model, with the exception that $x_j(t_i)+E_j(t_i)$ replaces $x_j(t_i)$ as the model-predicted value for Nef-negative cells. Simulations were begun 24 h prior to the first measurement assuming non-zero values of target cells and infectious virus at $t=-24$ h. The values of all other variables at that time, and the values of all variables prior to $t=-24$ h, were fixed at zero. The plotted curves show the best-fit of the eclipse model (lines) to the experimental data (points) for both target and eclipse cells (i.e., HSC-F Nef-negative T cells), infected cells, and the total and infectious viral load for the four different experiments conducted at different MOIs. We also calculated the dynamics of target and eclipse cells during our experiment separately (**gray and orange lines in top row**). The fitted values of the parameters and derived quantities, along with the initial ($t=-24$ h) values of target cells and infectious virus, are given in **Additional file 3.**
