# Supplementary material for: Quantification system for the viral dynamics of a highly pathogenic simian/human immunodeficiency virus based on an in vitro experiment and a mathematical model
Source: Retrovirology. 2012 Feb 25;9:18. doi: 10.1186/1742-4690-9-18 (PMC3305505; doi:10.1186/1742-4690-9-18)
Supplement: Additional file 3 — Table for estimated parameters in Additional files 1. Parameters values, initial values and derived quantities for the in vitro experiment with eclipse model. [file 1742-4690-9-18-S3.PDF]

Additional file 3. Parameters values, initial values and derived quantities for the *in vitro* experiment with eclipse model.

Parameter Name	Symbol	Unit	Value
Parameters obtained from simultaneous fit to full *in vitro* dataset			
Rate constant for infections	β_{50}	$(TCID_{50}/ml \cdot day)^{-1}$	6.0×10^{-5}
Decay rate of infected cells	a	day^{-1}	1.36
Production rate of total virus	k	RNA copies $\cdot$ day^{-1}	2.42×10^{4}
Production rate of infectious virus	k_{50}	$TCID_{50} \cdot day^{-1}$	0.23
Delay of Nef expression	τ_{ε}	hours	6.7
Quantities derived from fitted values			
Viral burst size (total)	k/a	RNA copies	1.78×10^{4}
Viral burst size (infectious)	k_{50}/a	$TCID_{50}$	0.17
Basic reproductive number (without removal)	R_0	—	68.1
Basic reproductive number (with removal)	R_0^*	—	7.65
Minimum fraction of infectious virus	k_{50}/k	$TCID_{50}$/RNA copies	9.50×10^{-6}

Variable	Unit	Fitted initial value at MOI of			
		2×10^{-3}	2×10^{-4}	2×10^{-5}	2×10^{-6}
$x_j(-24\ h)$	cells/ml	1.0×10^{7}	1.1×10^{7}	9.8×10^{6}	8.4×10^{6}
$v_{50j}(-24\ h)$	$TCID_{50}$/ml	19	2.2	0.82	0.51
