# Supplementary material for: Quantification system for the viral dynamics of a highly pathogenic simian/human immunodeficiency virus based on an in vitro experiment and a mathematical model
Source: Retrovirology. 2012 Feb 25;9:18. doi: 10.1186/1742-4690-9-18 (PMC3305505; doi:10.1186/1742-4690-9-18)
Supplement: Additional file 8 — Additional documentation for Additional files 4, 5, 6, 7. Detailed explanation of mathematical models used in Additional files 4, 5, 6, 7. [file 1742-4690-9-18-S8.PDF]

EXPLANATION OF ADDITIONAL FILE 4- 7.

Additional file 4-7. Fit of the mathematical model with various SSR^{W}s to experimental data of SHIV-KS661 *in vitro*. Here we used the following objective function:

$$SSR^{W} = \sum_{j=1}^{4}\left[\sum_{i=1}^{9}\left\{log\, x_j(t_i) - log\, x_j^{e}\,(t_i)\right\}^2 + \sum_{i=1}^{9}\left\{log\, y_j(t_i) - log\, y_j^{e}\,(t_i)\right\}^2 + \sum_{i=1}^{9}\left\{log\, v_{RNAj}(t_i) - log\, v_{RNAj}^{e}\,(t_i)\right\}^2 + W\sum_{i=1}^{9}\left\{log\, v_{50j}(t_i) - log\, v_{50j}^{e}\,(t_i)\right\}^2\right]$$

where *W* represent the weight between viral RNA and infectivity measurements in the fitting. In the case of *W=1*, the $SSR^{W}=SSR$. And the case (a), (b), (c), (d) are correspond to *W=0.0001*, *W=0.1*, *W=10*, *W=10000*, respectively. The curves show the best-fit of the model (Eqs.(5)-(8), lines) with various $SSR^{W}s$ to the experimental data (points) for the target cells, infected cells, and the total and infectious viral load for the four different experiments conducted at different MOIs. All data were fitted simultaneously as described in the text. The estimated parameters, the calculated original *SSR*, fitted *t=0* values of each quantity are given in **Additionla file 9**.
