# Supplementary material for: Quantification system for the viral dynamics of a highly pathogenic simian/human immunodeficiency virus based on an in vitro experiment and a mathematical model
Source: Retrovirology. 2012 Feb 25;9:18. doi: 10.1186/1742-4690-9-18 (PMC3305505; doi:10.1186/1742-4690-9-18)
Supplement: Additional file 9 — Table for estimated parameters in Additional files 4, 5, 6, 7. Parameters values and derived quantities for the in vitro experiment with various SSRWs. [file 1742-4690-9-18-S9.PDF]

Additional file 9. Parameters values and derived quantities for the *in vitro* experiment with various SSR^Ws.

Parameter Name	Symbol	Unit	Value				
			No weight	(a) 10000:1	(b) 10:1	(c) 1:10	(d) 1:10000
Parameters obtained from simultaneous fit to full *in vitro* dataset using weighted SSR^W							
Rate constant for infections	β_{50}	$(TCID_{50}/ml \cdot day)^{-1}$	4.95×10^{-5}	6.06×10^{-5}	4.79×10^{-5}	3.93×10^{-5}	2.09×10^{-5}
Decay rate of infected cells	a	day^{-1}	1.18	1.22	1.20	1.44	2.01
Production rate of total virus	k	RNA copies $\cdot$ day^{-1}	2.61×10^{4}	2.73×10^{4}	2.88×10^{4}	3.22×10^{4}	1.24×10^{5}
Production rate of infectious virus	k_{50}	$TCID_{50} \cdot day^{-1}$	0.22	0.19	0.26	0.26	0.28
Sum of squared residuals (SSR)	---	---	89.6	143.0	107.1	108.8	272.8
